# Roles of preoperative anxiety and depression in the outcomes of microvascular decompression in hemifacial spasm for adolescent patients

**DOI:** 10.1097/MD.0000000000026831

**Published:** 2021-08-13

**Authors:** Fan Wu, Pingcun Wei, Gang Wang, Changsong Wu, Yunlong Hu, Jinwang Hu

**Affiliations:** Department of Otolaryngology, Anhui No.2 Provincial People's Hospital, Hefei, Anhui Province, China.

**Keywords:** adolescent, anxiety, depression, hemifacial spasm, microvascular decompression

## Abstract

Hemifacial spasm (HFS) has been recognized as the frequently occurring disease of cranial nerve. At the same time, several articles indicate that, dystonia results in certain psychological disorders. Consequently, this study aimed to examine the association of preoperative depression and anxiety with HFS severity; meanwhile, the role in microvascular decompression (MVD) outcomes after surgery among adolescent patients was also examined.

All cases had been classified as two groups based on MVD outcomes among HFS cases; in addition, the preoperative Hamilton anxiety rating scale (HARS) and the Hamilton depression rating scale (HDRS) scores were compared between patients not and still suffering from spasm. Moreover, the multiple logistic regression model was employed in assessing the relationship between preoperative HARS as well as HDRS scores and outcomes of adolescent cases undergoing MVD.

The preoperative HARS and HDRS scores showed positive correlation with Cohen spasm grades in HFS patients. Meanwhile, compared with spasm-free group, patients of persistent spams group had apparently higher preoperative HARS and HDRS scores.

Our results suggest that, preoperative anxiety and depression status show close association with HFS severity, and they could also impact the MVD outcomes for adolescent cases.

## 1 Introduction

Hemifacial spasm (HFS), a rare neurological disorder classically described by Jannetta,^[1]^ has been featured by the intermittent and involuntary contractions of ipsilateral face muscles. Notably, HFS incidence is increasing, and it is reported to be as high as 7 to 15 in 100,000 individuals.^[2]^ Generally, HFS is defined to be a kind of serious facial dystonia, which will lead to serious persistent stress and negative effects.^[3,4]^ Spasm will occur in a higher frequency and persist for a longer time as HFS progresses, while this will potentially result in depression and anxiety, and eventually severely reduce the patient quality of life, particularly for the adolescents.^[5,6]^

Microvascular decompression (MVD) is deemed to be the first treatment selection for patients with the refractory neurovascular conflict.^[7,8]^ However, MVD Only mitigate spasm to some extent, alternatively, it fails in certain patients; besides, the annual recurrent rate is reported to be 1% to 9%.^[9,10]^ Consequently, it is still challenging to decrease the rate of relapse following MVD among HFS cases. At our center, patients afflicted with preoperative negative emotions are associated with a higher relapse rate or dismal outcomes following MVD. In some studies, HFS patients undergoing MVD have worse outcomes than those in older patients,^[11]^ and such a phenomenon is worth exploring. There are often negative emotions, such as anxiety and depression caused by various reasons, for adolescent patients.^[12]^ Some previous studies have reported the effect of preoperative anxiety and depression in patients with trigeminal neuralgia on the outcome of MVD, but no studies have been conducted on patients with HFS.^[13]^ As a result, the current work was conducted to examine the roles of preoperative anxiety and depression in the postoperative outcomes of MVD among HFS adolescent patients.

## 2 Materials and methods

### 2.1 HFS patients

The questionnaire results and medical records were collected retrospectively based on the new HFS adolescents that received MVD in Department of Otolaryngology, Anhui No.2 Provincial People's Hospital between September 2010 and September 2019. Notably, cases conforming to the following standards were enrolled in this study:

1. HFS cases with primary HFS;
2. HFS patients that had not received non-drug HFS treatment before surgery, including MVD, radio-frequency ablation and stereotactic radiosurgery;
3. HFS cases that had no psychologic disease;
4. HFS cases not taking any psychedelic or hypnotic; and
5. the HFS cases who submitted informed consent for study participation.

Our study protocol was approved by the Institutional Ethics Committee of Anhui No.2 Provincial People's Hospital.

### 2.2 Assessment of outcomes

Demographical as well as clinicopathological features of all collected cases were extracted based on the questionnaires and medical records, such as age, gender, duration, education, and Cohen spasm grades (0–4, Grade 0: no spasm; Grade 1: blink increase or slight facial muscle fibrillation caused by external stimulation; Grade 2: spontaneous slight palpebral and facial muscle twitching without functional disturbance; Grade 3: obvious spasm with slight dysfunction; Grade 4: severe spasm and dysfunction). At the same time, anxiety and depression assessments were collected based on the HARS score (0–7: normal, and >7: related to depression; besides, a greater score was indicative of more severe depression), together with HDRS score before surgery. In addition, HFS cases had also been classified as two groups based on postoperative MVD outcomes, among them, patients suffering from spasm for 6 months postoperatively were classified as persistent spasm group, whereas cases with no spasm recurrence had been classified as spasm-free group.

### 2.3 Statistical methods

The statistical analysis was carried out using the R software (http://www.R-project.org) and the Empower(R) software (www.empowerstats.com, X&Y solutions, Inc Boston, MA). At first, all variables were examined by Kolmogorov–Smirnov test to see whether they conformed to normal distribution. Afterwards, the normally distributed data were assessed by one-way analysis of variance (ANOVA) or two-tailed Student's *t* test. Meanwhile, various groups were compared for non-parametric data by Mann–Whitney *U* test. Moreover, the relationships between anxiety as well as depression and postoperative outcomes were examined through the multiple logistic regression model. Associations between HARS as well as HDRS scores and the Cohen spasm grades would be examined using Pearson's correlation coefficient test.

## 3 Results

### 3.1 Objects of study

Altogether 86 HFS cases, including 48 women (55.8%) and 38 men (44.2%) had been recruited for eventual study analysis. Table 1 displays the demographic data for each subject. The age range of HFS cases was 10 to 21 years, with the median of 13 years. There were 70 and 16 cases in the spasm-free and persistent spasm groups, respectively.

**Table 1:** Comparison between spasm-free patients and those with persistent spasm.

Outcome variable	Persistent spasm	Spasm-free	*P*
No.	16	70	
Age (years)	16.2 ± 20.0	15.9 ± 12.8	.92
HDRS score	14.3 ± 5.4	6.5 ± 3.4	<.01
HARS score	12.8 ± 4.1	6.4 ± 4.0	<.01
Duration (years)	3.0 ± 3.7	3.2 ± 4.0	.83
Gender			.96
Male	7 (43.8%)	31 (44.3%)	
Female	9 (56.2%)	39 (55.7%)	
Side			.84
Left	8 (50.0%)	37 (52.9%)	
Right	8 (50.0%)	33 (47.1%)	
Grades of Cohen			.09
II	4 (25.0%)	30 (42.9%)	
III	5 (31.2%)	27 (38.6%)	
IV	7 (43.8%)	13 (18.6%)	
Educational level			.39
Primary	2 (12.5%)	9 (12.9%)	
Junior high school	4 (25.0%)	31 (44.3%)	
Senior high school	7 (43.8%)	17 (24.3%)	
College	3 (18.8%)	13 (18.6%)	

### 3.2 Roles of anxiety and depression in postoperative MVD outcomes for HFS cases

**Figure 1:**
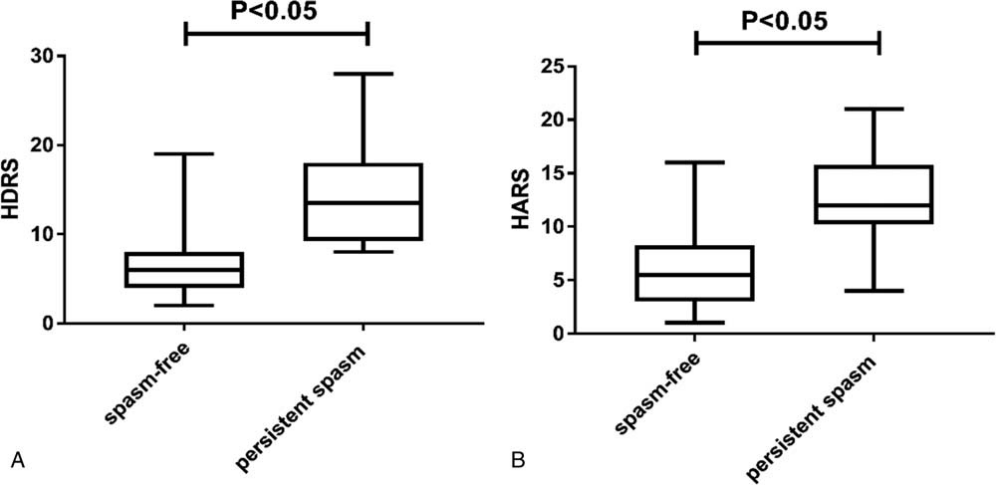
Comparison preoperative HDRS and HARS score between persistent spasm patients and spasm-free patients.

**Figure 2:**
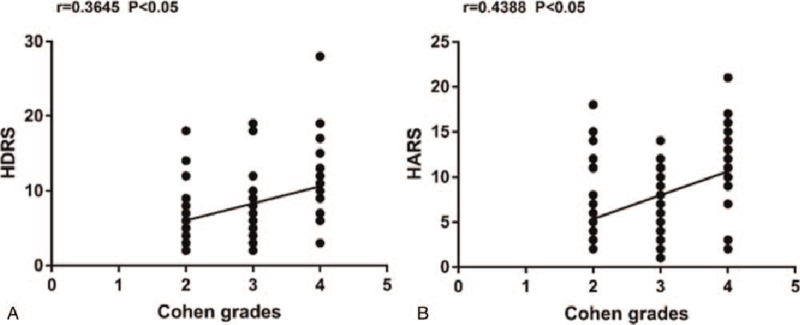
Correlations of Cohen spasm grades with preoperative HDRS and HARS scores in HFS patients.

Figure 1 suggested that, preoperative HARS and HDRS scores were apparently increased for persistent spasm group compared with those for spasm-free group. Multivariate analysis was carried out to further evaluate such relationship (Table 2). Clearly, the results showed that, preoperative HARS and HDRS scores displayed notable correlation with the postoperative outcomes. Therefore, anxiety and depression could serve as the risk factors for dismal MVD outcomes. With regard to the associations between anxiety as well as depression and HFS severity, HARS and HDRS scores showed positive correlation with Cohen spasm grades among HFS adolescents (Fig. 2).

**Table 2:** Multivariate regression showing the effect of preoperative HDRS and HARS scores on the outcome after MVD.

	Non-adjusted		Model I		Model II	
	OR (95% CI)	*P*	OR (95% CI)	*P*	OR (95% CI)	*P*
HARS	1.2 (1.1, 1.5)	<.01	1.2 (1.1, 1.5)	<.01	1.3 (1.0, 1.5)	<.01
HDRS	1.2 (1.0, 1.4)	<.01	1.2 (1.0, 1.4)	<.01	1.2 (1.0, 1.4)	<.01

## 4 Discussion

HFS leads to segmental myoclonus, while this will then lead to psychological and physical distress, thereby reducing patient quality of life.^[3,7,8]^ In recent years, the effective rate of MVD in young HFS patients is decreased relative to that among older cases. The exact cause of the efficacy among young patients is confusing, and it may be related to the special psychological state of adolescents. A total of 86 HFS adolescents receiving one single MVD treatment were enrolled in this study, so as to analyze depression and anxiety in the remaining HFS.

Adolescence is associated with great concerns during general mental health development, including antisocial behavioral problems and depressive symptoms. Typically, burden related to the occurrence rate of such behaviors and concerns is usually cited to be a leading factor inducing adolescent disability and mortality.^[14,15]^ Depressive and anxiety diseases have affected about 5% to 7% adolescents in the world annually.^[16,17]^ Therefore, the preoperative psychological state of HFS adolescents is worthy of attention. The association of the preoperative psychological status in adolescents with postoperative MVD outcomes has not been reported yet.

According to our findings, anxiety and depression could potentially serve as risk factors indicating dismal MVD outcomes for adolescent HFS cases, and such result was consistent with those in other studies on cranial nerve disease.^[13,18]^ Anxiety and depression have negative effect on relevant physiopathological mechanism that affects the treatment course for disease, and this will then affect patient medical conditions, which was related to hypertarachia of certain biological pathways as well as depression of central nervous system (CNS).^[5,19]^ In our study, anxiety and depression showed close correlation with HFS severity; therefore, it was recommended that the collaboration between the neurosurgery departments and psychiatric teams should be increased to assess HDRS and HARS among HFS cases. Consequently, the first psychiatric assessment and the appropriate treatment (when necessary) must be enrolled for such patients before the first surgery. The emotions in patients with potential anxiety or depression can be regulated by means of medication or psychological treatment, which further enhances the MVD efficacy.

A large number of recent studies have investigated the psychological state as well as the psychological intervention for perioperative patients.^[20,21]^ Importantly, using suitable treatments to improve depression and anxiety among patients is of crucial importance. As reported in some studies, music therapy and relaxation training show positive effect on the psychological state among adolescents; in addition, cases undergoing coping training, such as positive self-talk, relaxation and deep breathing had decreased anxiety and pain severity.^[22]^ Moreover, psychological intervention can also mitigate perioperative anxiety in these cases. Specially, complicated surgical procedures and equipment will be applied to patients preoperatively, and patients are encouraged to take these treatments. Additionally, successful operation cases can also be described to patients to boost their confidence, particularly for the teenagers who are full of fear of unknown things.^[22–24]^

However, there were some limitations of the current study. First, only a small sample size was enrolled, and the influences of various psychological factors on remaining HFS following one single MVD could not be distinguished. Secondly, the precise temporal association of HFS onset with depression and anxiety onset could not be evaluated. Thirdly, selection bias could not be eliminated due to the lack of the prospective method, as well as randomization by the long-term follow-up.

## 5 Conclusions

Our results demonstrate that, preoperative anxiety and depression show close correlation with the severity of HFS, which may result in dismal MVD outcomes. Our results can shed new light on the effect of perioperative psychological intervention on improving HFS adolescent prognosis following MVD.

## Acknowledgments

The authors would like to thank all the individuals who offered help and advice on this study.

## Author contributions

**Conceptualization:** Fan Wu, Jinwang Hu.

**Funding acquisition:** Jinwang Hu.

**Investigation:** Changsong Wu, Yunlong Hu.

**Methodology:** Fan Wu, Pingcun Wei, Gang Wang, Jinwang Hu.

**Supervision:** Jinwang Hu.

**Validation:** Jinwang Hu.

**Visualization:** Fan Wu.

**Writing – original draft:** Fan Wu.

**Writing – review & editing:** Fan Wu, Jinwang Hu.
